# Development of the Self Optimising Kohonen Index Network (SKiNET) for Raman Spectroscopy Based Detection of Anatomical Eye Tissue

**DOI:** 10.1038/s41598-019-47205-5

**Published:** 2019-07-25

**Authors:** Carl Banbury, Richard Mason, Iain Styles, Neil Eisenstein, Michael Clancy, Antonio Belli, Ann Logan, Pola Goldberg Oppenheimer

**Affiliations:** 1 Chemical Engineering, University of Birmingham, Birmingham, UK; 2 Physics and Astronomy, University of Birmingham, Birmingham, UK; 3 Computer Science, University of Birmingham, Birmingham, UK; 4 Institute of Inflammation and Ageing, University of Birmingham, Birmingham, UK

**Keywords:** Raman spectroscopy, Scientific data, Nanophotonics and plasmonics

## Abstract

Raman spectroscopy shows promise as a tool for timely diagnostics via *in-vivo* spectroscopy of the eye, for a number of ophthalmic diseases. By measuring the inelastic scattering of light, Raman spectroscopy is able to reveal detailed chemical characteristics, but is an inherently weak effect resulting in noisy complex signal, which is often difficult to analyse. Here, we embraced that noise to develop the self-optimising Kohonen index network (SKiNET), and provide a generic framework for multivariate analysis that simultaneously provides dimensionality reduction, feature extraction and multi-class classification as part of a seamless interface. The method was tested by classification of anatomical *ex-vivo* eye tissue segments from porcine eyes, yielding an accuracy >93% across 5 tissue types. Unlike traditional packages, the method performs data analysis directly in the web browser through modern web and cloud technologies as an open source extendable web app. The unprecedented accuracy and clarity of the SKiNET methodology has the potential to revolutionise the use of Raman spectroscopy for *in-vivo* applications.

## Introduction

**Figure 1:**
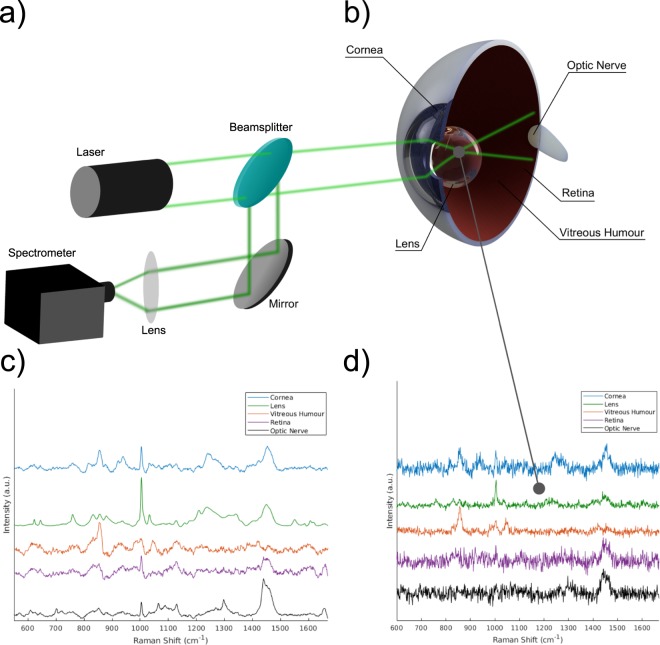
(**a**) Schematic of a typical Raman setup. Light from a laser is focused into the eye. Backscattered light is then directed via a beamsplitter to a spectrometer. (**b**) Schematic of the eye. (**c**) Averaged Raman spectra from isolated tissue segments of each anatomical layer. (**d**) Typical raw spectra for each tissue type used for training and classification.

Raman spectroscopy is a non-invasive technique for immediate detection and analyses of the biochemical composition of analytes by measurement of the inelastic scattering of light. A schematic showing a typical experimental arrangement is shown in Fig. 1a, where longer wavelength inelastically scattered light from the sample is directed to a spectrometer via a beamsplitter. It is one of most sensitive optical spectroscopy methods yet can be packaged as a hand-held device^1,2^. Therefore, there is a considerable interest in applying Raman spectroscopy for the point-of-care detection of clinical biomarkers. Ophthalmic applications have received particular interest, as the optically clear nature of the eye provides a convenient route for *in-vivo* measurements^3–8^.

The eye consists of a number of anatomical layers (Fig. 1b), each with their own specific functions, which are biologically and chemically distinct. Despite studies highlighting the potential for early diagnostics of diseases that target a specific tissue type, there is currently no direct comparison of Raman spectra from each anatomical tissue layer. Whilst Raman spectroscopy offers excellent chemical specificity, biological samples form complex permutations built from only a few amino acid building blocks, resulting in considerable spectral overlap and complex data analysis^9^. The problem is further compounded by poor signal to noise as a result of the Raman effect being relatively weak. Particularly for diagnostic applications, it is crucial to be able to accurately identify and understand the signal originating from different parts of the eye. In addition to eye tissue, the optic nerve was included as an additional class, as this represents a particularly interesting target for applications beyond ophthalmology. Forming part of the central nervous system, the optic nerve is technically part of the brain and lies at the same focal plane as the retina. The ability to spectrally isolate and characterise the optic nerve from the rest of the eye would lay foundations for further diagnostic possibilities of major neurological diseases including for instance: traumatic brain injury, multiple sclerosis or Alzheimer’s disease.

The analysis of such datasets is often conducted as a workflow of three stages: projection, feature extraction and classification. The initial step (projection) aims to show spatial separation of data from spectra according to different types or classes in two or three dimensions. Feature extraction then follows, with the aim of understanding what Raman bands in the data cause any separation observed in the projection step. Finally, this information is used to build a classification model, that can make accurate predictions about future unlabelled data.

In the field of Raman spectroscopy and even more generally in chemometrics, principal component analysis (PCA) is favoured for projection and feature extraction, followed by partial least squares discriminant analysis (PLS-DA) and more recently deep learning models for classification^10–12^. However, PCA routinely shows poorly defined class boundaries, struggles with large intra-class variance (such as biological samples) and quickly breaks down for multi-class problems^13^. Furthermore, classification is often handled in isolation to projection and feature extraction, forming an semantic disconnect, and whilst deep learning has shown impressive classification results, these methods offer no insight into the underlying physical and chemical changes.

Our aim is to provide a single method to address each of these stages, connected by a single mathematical principle and improve on the issues found using PCA based approaches. Work by Brereton *et al*. highlighted the use of self organising maps (SOMs) applied to nuclear magnetic resonance spectroscopy in comparison to PCA, and showed much clearer visualisations. The work was further extended to support feature extraction and classification using SOMs by the introduction of the self organising map discriminant index (SOMDI)^14–16^.

Here, we develop an improved SOMDI based supervised learning method, defined as the self-optimising Kohonen index network (SKiNET) to demonstrate effective classification, and illustrate the complete linked workflow from projection to classification by means of a user-friendly web app^17^. This represents a major shift, that follows a growing trend in industry to move from traditional desktop applications to the cloud (including office suites, multimedia editing and computer aided design (CAD)) and yet the advantages of connected scalable applications are seldom leveraged in the scientific community.

The SOM or Kohonen map was first described by Teuvo Kohonen in 1982 as a model inspired by nature and the way that neurons in the visual cortex are spatially organised according to the type of visual stimuli^18^. The SOM defines a 2D map of neurons, typically arranged as a grid of hexagons. Each neuron is assigned a weight vector, which is initialised randomly and has a length equal to the number of variables in a spectrum. The weight vector effects which neuron will be activated for a given sample and neighbouring neurons will have similar weights. Spatial clustering is therefore observed in the trained map, with spectra that exhibit distinct properties activating different neurons. In order to understand which features in the data cause certain neurons to activate over others, the self organising map discriminant index (SOMDI) was used^15^. The SOMDI introduces class vectors as labels for each spectrum and corresponding weight vectors for each neuron, without influencing the training process. These allow for the identification of what type of data a given neuron activates, which can be used to inspect the weights across all neurons and extract prominent features belonging to each class.

## Results

Raman spectra were randomly sampled from tissue segments from 11 separate enucleated eyes, by acquiring coarse map scans of 88 spectra per tissue segment. The aqueous humour sitting between the cornea and crystalline lens, consisting mostly of water, was neglected. Figure 1c shows averaged spectra representative of each tissue type, or class to be identified. Individual Raman spectra were kept consciously noisy by using a short acquisition time and limited laser power, to be representative of real world applications, which are limited by both scan time and maximum permissible exposure (MPE) defining eye safe limits^19^. Examples of typical raw spectra (after cosmic ray removal and baseline subtraction) are shown in Fig. 1d. Whilst the averaged spectra across each class showed obvious spectral differences, a large degree of variance was seen across each map scan (Supplementary Information, Fig. S1). As neural networks are data hungry algorithms by nature, it was hypothesised that a meaningful model could be trained by using a large enough number of noisy inputs. Initially, a 25% partition from each class of the 4840 spectra were reserved for test data.

Our results are presented as a typical mutlivariate analysis workflow of: (1) projection of the hyperspectral data set into 2D space; (2) feature extraction to identify which spectral bands are characteristic of each tissue type and (3) a classification model to automatically identify the origin of an unknown spectrum. In each case, the SOM shows dramatic improvement over PCA based methods, offering better presentation of the data, clearer insights and greater classification accuracy.

### Data projection

**Figure 2:**
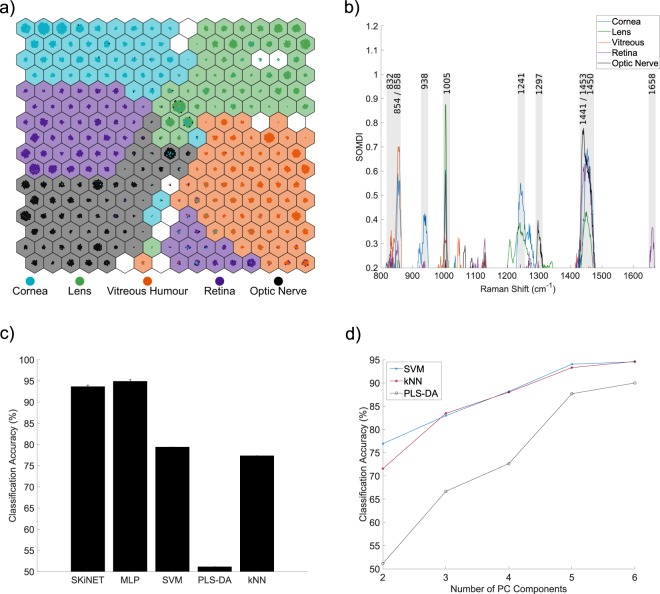
(**a**) SOM trained on spectra across the 5 eye tissue types. (**b**) SOMDI showing relative importance of different bands for each class to observed clustering in the SOM. (**c**) Classification accuracy of tissue using SKiNET against current state-of-the-art (multi-layer perceptrons (MLP), support vector machines (SVM), partial least squares discriminant analysis (PLS-DA) and k-nearest neighbours (kNN)). (**d**) Effect of number of principal components on classification accuracy for PCA based methods.

Figure 2a shows a clear separation of the data from the five tissue classes arranged as a 16 × 16 SOM, trained on spectra from the five tissue classes. Neurons (hexagons) are coloured according to the modal class they activate, from the training set of Raman spectra. Neurons that have no majority class or activate non of the training data are shown in white. Coloured circles within each neuron represent spectra from the training data that have been activated for that neuron. To aid visualisation, circles have been forced to not overlap in space using the D3-force library^20^, providing an alternative mechanism to display sample frequency and class overlap for each neuron. Note that almost all of the available white space in the figure is used completely. For each class, there is a clearly defined block of neurons, with many of these activating only a single tissue type. An approximately even distribution in the number of neurons required to identify each class is observed, with a slightly higher weighting for the vitreous humour. As a result of the vitreous humour consisting mainly of water and containing very few cells, the additional effort required by the network to isolate the tissue can be observed in the map. This can be considered by analogy to how the brain associates a larger number of neurons to facial features, than for example arms and legs (the cortical homunculus).

The majority of poorly separated samples are located centrally at the boundary between classes and extend down to the bottom edge of the map. Interestingly, in this region, there is a cluster of samples predominately corresponding to the retina, indicating that a number of retina samples are particularly noisy, further corroborated by being spatially located near other neurons that also lack any well defined class. While the SOM is analogous to the PCA scores plot (Supplementary Information, Fig. S2a), PCA performs particularly badly when compared against the SOM. However, it should be noted that the level of separation observed by PCA is completely inline with results commonly reported in the literature. Since PCA relies on separation by variance in the data, the class clusters are bound around a central point, as a result of noise or absence or spectral features, causing significant spatial overlap.

### Feature extraction

The SOMDI provides a representation of weights associated with neurons that identify a particular class. A higher SOMDI intensity indicates a greater importance of particular inverse centimetres along the x-axis of a spectrum. Figure 2b shows the SOMDI overlaid for each class, where the most important Raman bands associated with each tissue layer can be easily identified. Despite the level of noise in the original data, well defined peaks are resolved in Fig. 2b, which are either more prominent or unique to each class. Strong weights are attributed to the cornea at 938 (C-C stretch) and 1241 cm^−1^ (C-N stretch), which also correspond, with a certain confidence, to the stretching modes of the C-C backbone and amide III modes of collagen.

The crystalline lens of the eye is predominately identified by a very strong SOMDI weight at 1005 cm^−1^ (2,4,6C radial) and is attributed to phenylalanine, which is abundant in water-soluble proteins present in the lens and directly relates to the tissue’s function. The high polarizability of this molecule, which results in a large Raman scattering cross-section, aids in increasing the refractive index of the lens thus, providing fine focusing of light onto the retina. The vitreous humour is more challenging to isolate, with the strongest weights at 854, 858 cm^−1^ overlapping with significant weights for cornea, which have been associated with proline in collagen, along with small distinct weights at 832, 1044 and 1049 cm^−1^. These bands may be indicative of the difference in collagen type found in the cornea versus the vitreous humour (type I vs. type II respectively) yet, a direct comparison of the two protein types is further required to support this postulation. The interpretation and discrimination of collagen types by Raman spectroscopy is currently an active area of interest, where SkiNET may also offer additional insight^21,22^.

The remaining two classes of retina and the optic nerve are perhaps the most intriguing, located within the same focal plane, with the optic nerve connecting directly to the brain. An isolated peak at 1658 cm^−1^ (C=O stretch) identifies the retina and is associated with amide I (α-helix) groups in proteins. The detection of light by rods and cones in turn, relies on photo-receptive proteins known as opsins, which have an α-helical secondary structure. In contrast, the optic nerve can be characterised by a strong weight at 1441 (CH2 scissoring, CH3 bending) and 1297 (CH2 deformations) cm^−1^, strongly associated with lipids and fatty acids. The brain is composed of nearly 60% fat, with lipids and fatty acids playing important roles in brain function, which here we observe as a clear marker for the distinction between brain and eye tissue via Raman spectroscopy^23^. Furthermore, the optic nerve is devoid of photo-receptive cells and responsible for the blind spot in humans and therefore, the peaks at 1441 and 1658 cm^−1^ act as biologically relevant markers for each^24^. Individual bond assignments were made with reference to Larkin^25^, and associations to high level biological structures based on the work by Talari *et al*. and Movasaghi *et al*., providing databases of Raman bands found in biological tissue^26,27^.

Finally, unlike PCA loadings, which are often used to show similar information, the SOMDI can be interpreted in isolation. Conversely, PCA loadings are only relevant to a direction in PC space, relying on constant reference to the scores plot, which quickly become cumbersome for multi-class problems or where multiple PC scores are considered (Supplementary Information, Fig. S2b).

### Classification

Automated classification of Raman spectra and assignment to a particular tissue type or disease state is perhaps the most important step for the translation of Raman-based diagnostic techniques to real-world, clinical applications. However, whilst SOMs have historically been used for visual separation of data, experimental results of classification are rare. The most common method is to look-up the modal class of the neuron activated for a test sample, as used to colour neurons in Fig. 2a. Since the SOMDI automatically provides class labels, the maximum SOMDI weight can also be used to perform class identification of any given neuron. However, both of these methods remain unsupervised learning mechanisms, without optimisation towards the correct answer in the training set. This is in contrast to widely used supervised learning algorithms, such as multi-layer perceptrons (MLP), support vector machines (SVM), partial least squares discriminant analysis (PLS-DA) and k-nearest neighbours (kNN)^28–30^.

Supervised learning can be introduced to SOMs by allowing the class weights used for the SOMDI to influence the learning process. For large enough label values, this effectively forces the map to cluster, however can result in over-fitting^16^. For our data, no benefit was observed using this method over the modal class on the unsupervised SOM (Supplementary Information, Fig. S3). Instead, a concept from learning vector quantisation (LVQ) was applied to the trained map and defined as a self-optimising Kohonen index network (SKiNET). A penalty is introduced for spectra (from the training set) that activate neurons identifying a different class. This has a natural tendency to self-optimise, with the identical behaviour to the vanilla SOM when training data activate the correct class.

Figure 2c shows the classification accuracy across all five tissue types using SKiNET, vs. current state-of-the-art methods. A 25% partition of the original data set was randomly assigned as test data and not used for training and optimisation of the network. The remaining 75% was used to optimise hyper-parameters of each classifier, which were tuned by performing 10-fold stratified cross validation. Most notable is the considerable improvement over PLS-DA, which is perhaps the most widely adopted method in chemometrics^31^. PCA was used as a dimensionality reduction method prior to classification for SVM, PLS-DA and kNN. It should be emphasised that only the first two principal components were kept. Figure 2d shows that by including a larger number of components, each of the classification methods can achieve a similar accuracy. The case of keeping more components for classification than are used for projection and feature extraction is routinely used in the literature. The alternative is to show several pairwise PCA scores plots, which arguably leaves the data in a high dimensional space^10,11,32^.

However, by implementing SKiNET we are able to achieve a classification accuracy equivalent to keeping 6 components, whilst still being able to fully separate the data in only two spatial dimensions; equivalent to using 2 PCA components. Additionally, SKiNET showed a comparable performance to multi-layered perceptrons (MLP), whilst providing clear visualisations and feature extraction that MLPs and other neural network based methods lack. The confusion matrix (Supplementary Information, Table. S1) provides a breakdown of test samples classified into each class, and highlights the stability of the method across each of the five tissue types.

## Discussion

The use of spectral fingerprints for clinical diagnostics requires two major components: the ability to quickly and accurately distinguish between different states (such as tissue types or diseases) and an understanding of the underlying chemical differences that enable such separation. The former is driven by an obvious need to perform timely diagnostics, but these decisions must be underpinned by biologically relevant changes. These issues are usually treated in isolation by multivariate techniques, with the best classification methods providing no insight into their nature. SKiNET addresses this disconnect, by using a single, simple architecture to provide clear visualisations and a high classification accuracy, whilst retaining an understanding of the major chemical differences between classes. Furthermore, the SOM removes the need for much of the linear algebra and matrix notation required to fully appreciate PCA. Instead, the SOM can be adequately described using only addition and subtraction.

We reiterate that SOMs can offer a vastly superior spatial separation of chemometric data, that has now been demonstrated for both NMR and Raman spectroscopy. The SOM can be considered mathematically as a non-linear equivalent to PCA, and therefore hints that these data may not in fact be linearly separable, as would normally be assumed from Raman spectroscopy and is a requirement for PCA to be valid^30^. Our assertion is that the inherent heterogeneity combined with spectral overlap could easily lead to this condition for biological samples. Despite the level of overlap and noise present in our raw data, the SOMDI offers a convenient method to quickly isolate important bands and automatically act as a noise filter. By using the SOMDI it was possible to easily identify prominent markers for bulk tissue properties in each of the tissue types considered.

LVQ offers a convenient means of introducing supervised learning into the SOM, however there are several variations of the LVQ algorithm that have not been explored here. This remains an area for future work, in addition to automatically setting the map parameters such as number of neurons, neighbourhood size, and an adaptive learning rate. Finally, it was shown that SOMDI weights could act as iterative class labels that are present throughout the learning process and change dynamically. As a result, there is scope to explore SKiNET based classification in conjunction with other SOM optimisation methods, that presently rely on a hit count (majority voting), which requires placing all of the training data into the SOM at every learning step where we wish to identify the winning class for a given neuron^33^. Since the SOMDI provides a constant dynamic neuron identifier, this would allow for scaling to larger training sets using such methods.

In general, SKiNET was seen to offer a huge classification improvement over existing methods, performing particularly better than PLS-DA, which is the current *status quo* in chemometrics. Several of the points stressed here have been mentioned in other publications across different disciplines, but never cohesively. It is therefore of equal importance that the entry point for SKiNET is not to download, buy a software package or compile scripts; but simply visit a website and upload data.

The ability to quickly identify tissue from the noisy spectral response of a short acquisition, as demonstrated here represents an important stepping stone towards the practical applicability of *in-vivo* ophthalmic Raman spectroscopy, allowing for the capture of clean signal in the region of interest only. Filtered signal could then be fed into a second SKiNET model designed to distinguish between specific disease states.

## Methods

### Self-optimising kohonen index network (SKiNET)

The SOM is represented by a set of neurons arranged in a (hexagonal) grid. Here, we describe the basic SOM algorithm with SOMDI variables added for feature extraction^15,18^. We then describe how LVQ is included as an additional step to provide supervised learning, whilst using the SOMDI to identify each neuron class. Variables definitions are shown in Table 1 for reference. In each case, the capitalised letter represents the set for a given variable, e.g., the SOM contains a grid of *N* neurons.

**Table 1:** Definitions of variables used to describe SOM and SKiNET.

Variable	Description	Length
*i*	A single spectrum	1015
*j*	Spectrum class label vector	5
*s*	Training sample and label	[*i*, *j*]
*n*	A neuron	
*w*	Spectrum weight vector	length(*i*)
*c*	Class weight vector	length(*j*)
*t*	Training step	integer

Initially, every neuron is assigned weight vectors *w* (spectrum weight) and *c* (class weight), which are randomly initialised. The SOM is then trained according to the following algorithm:

1. Select a sample *s* at random from *S*
2. Calculate the euclidean distance, *d* for each *n*:
  $$d=\sqrt{{i}^{2}+{w}^{2}}$$
3. Define the best matching unit (*BMU*) as the neuron with minimum *d*
4. Update weights, *w* and *c* of each neuron be similar to the input:

$$scaleFactor=neighbourhood(BMU,t)\ast learningRate(t)$$

$$w=w+scaleFactor\ast (i-w)$$

$$c=c+scaleFactor\ast (j-c)$$

The map is gradually trained by repeating these steps numerous times. The update step applied in step 4 depends on a *neighbourhood* function which ensures neurons closest to the BMU are effected most (according to a Gaussian function), with a decreasing neighbourhood size with each *t*. Secondly a *learningRate* influences the update criteria, which linearly decreases with each iteration, *t* from a fixed initial starting value. To note, while class weights are updated in steps 4, they play no role in step 2, i.e., the spectra alone are responsible for finding the BMU.

**Figure 3:**
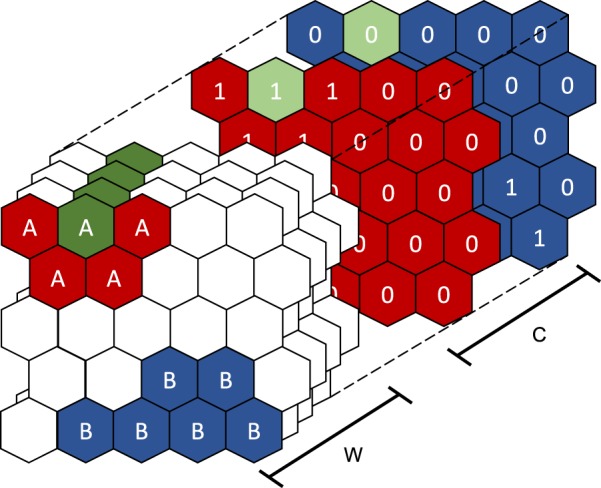
Illustrative example of SOM for two classes A and B, coloured red and blue, respectively. The weight vectors *W* and *C* can be thought of as making up additional planes in the z direction. Class planes are formed having values close to 1 for a given class and values close to 0 otherwise. These are used for classification and identification of the most important planes in *W* for the SOMDI.

The class vectors *J* have values of 1 for a given index or otherwise 0, e.g.,[1, 0, 0, 0, 0] and [0, 1, 0, 0, 0] representing labels for two of the five classes. As the map is trained, the neuron class vectors *C* become close to 1 as the neuron activates more of one spectral type and tend towards zero for all other class variables. Figure 3 illustrates how these vectors define class planes that are used to form the SOMDI. Once the map is trained, the class of any given *n* can be identified by finding the maximum of *c*.

### Supervised learning

A second learning round is then applied, keeping the spatial mapping of neurons, but changing the update criteria to use rules from LVQ:

1. Start with trained SOM
2. Select a sample *s* at random from *S*
3. Calculate euclidean distance, for each *n*
4. Define *BMU* as the neuron with minimum *d*
5. Identify *BMU* and *s* class labels:
  $$clas{s}_{j}=indexOf(max(j))$$
  $$clas{s}_{c}=indexOf(max(c))$$
6. Update *BMU w* and *c*:

if (*class*_*j*_ = *class*_*c*_) then

$$w=w+scaleFactor\ast (i-w)$$

$$c=c+scaleFactor\ast (j-c)$$

else

$$w=w-scaleFactor\ast (i-w)$$

$$c=c-scaleFactor\ast (j-c)$$

where only the update step changes when *s* lands on an incorrect neuron, to move both the spectrum weights and class weights of the BMU further away (and so making the neuron less likely to activate a similar spectrum in future iterations). During LVQ only the the BMU is updated under this regime and thus, represents only a small perturbation to the network. By analogy, this can be thought of as applying fine details to a painting, after the initial broad brush strokes to block in colours.

The method is described as self-optimising, since when the BMU class matches that of the input, the BMU weights are moved closer to the input as per the original unsupervised SOM algorithm. This allows a natural optimum to be reached, whilst preventing over-fitting. A second consequence of SKiNET, is a greater degree of freedom for each neuron. In the update step, the weight vector for the data and class labels are both updated, allowing for the class definition of a neuron to dynamically change as the map is trained.

### Samples

Tissue samples were retrieved within hours of slaughter from a total of 11 enucleated porcine eyes, provided by Rowley CH Ltd, a local abattoir. Eyes were dissected to isolate small segments of cornea, lens, vitreous humour, retina and optic nerve. Tissue samples were prepared using a protocol suggested by Cui *et al*., using glass slides covered with aluminium foil as a cost effective substrate, and allowed to air dry for 24 hours^34^.

### Raman spectroscopy

An InVia Qontor (Renishaw plc) equipped with a 785 nm laser was used for all measurements. LiveTrack maps over a sample area of 110 × 77 microns were acquired for each sample, with an acquisition time of 5 s for each point location in the map, and laser power of 2 mW, a 50 × Leica objective (0.75 NA), 1200 l/mm grating with scans recorded in the range 550–1670 cm^−1^. A total of 88 scans per tissue sample were recorded (4840 spectra total).

### Software and preprocessing

Baseline subtraction and cosmic ray removal were applied in WiRE 5.1 (Renishaw plc), each sample was independently standardised by mean centering and scaling to unit variance using Scikit-learn in python^35^. The package was then used to define training/test partitions, cross validation folds and define models for each classifier. The SOM based methods were defined in JavaScript by forking an existing open source SOM library^36^. The entire library was heavily refactored to include support for SKiNET, and is available on Github^37^. For consistency, a wrapper was created around the JavaScript library, to expose the same methods in python, allowing for all models to be benchmarked via the same script.

## Supplementary information

Supp Info

## Data Availability

For SOM and SOM based classification, the code was implemented in JavaScript, chosen for it’s ubiquity on almost every modern device. This allowed for the creation of simple, user friendly web interface that can be easily accessed from any location, without any need to install or compile a single line of code. The lack of easily accessible tools has previously been cited as a reason for poor adoption of such methods as seen in chemometrics. We are aiming to address this gap by providing both a library and web app available as open source tools^17,37^. All Raman spectra used in the analysis presented are available in electronic form from the corresponding author upon request.
