# Supplementary material for: Development of the Self Optimising Kohonen Index Network (SKiNET) for Raman Spectroscopy Based Detection of Anatomical Eye Tissue
Source: Sci Rep. 2019 Jul 25;9:10812. doi: 10.1038/s41598-019-47205-5 (PMC6658481; doi:10.1038/s41598-019-47205-5)
Supplement: Supplementary file 1 — Supp Info [file 41598_2019_47205_MOESM1_ESM.pdf]

Development of the Self Optimising Kohonen Index Network (SKiNET) for Raman Spectroscopy Based Detection of Anatomical Eye Tissue

Carl Banbury[1] Richard Mason[2] Iain Styles[3] Neil Eisenstein[1]
Michael Clancy[1] Antonio Belli[4] Ann Logan[4]
†Pola Goldberg Oppenheimer[1]

[1]*Chemical Engineering, University of Birmingham* [2]*Physics and Astronomy, University of Birmingham* [3]*Computer Science, University of Birmingham* [4]*Institute of Inflammation and Ageing, University of Birmingham*

Supplementary Information

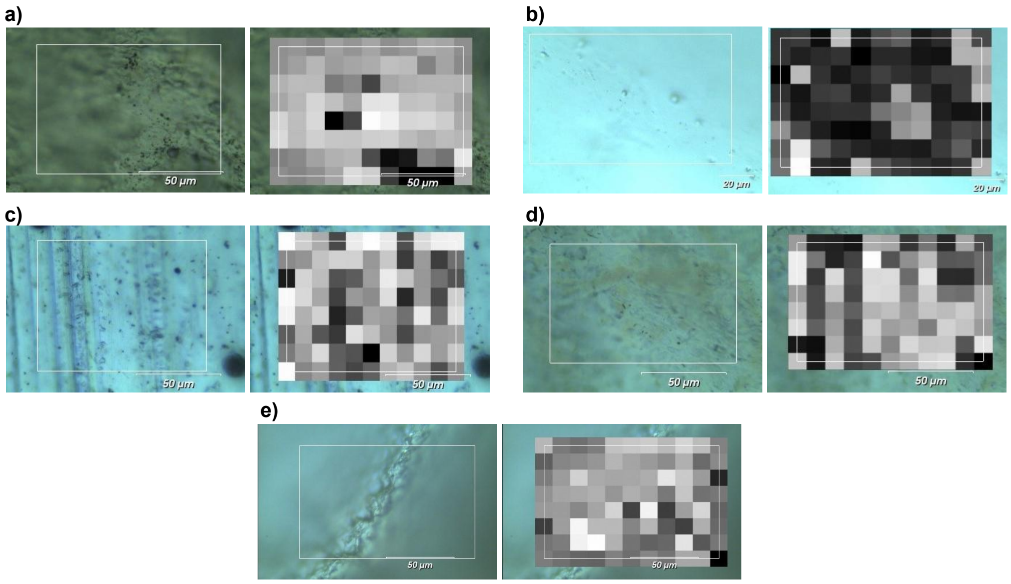

Figure S1: Paired examples of bright field optical microscope images (left) and PCA scores across map scan (right) for each tissue type: **a**, cornea, **b**, lens, **c**, vitreous humour, **d**, retina and **e**, optic nerve.

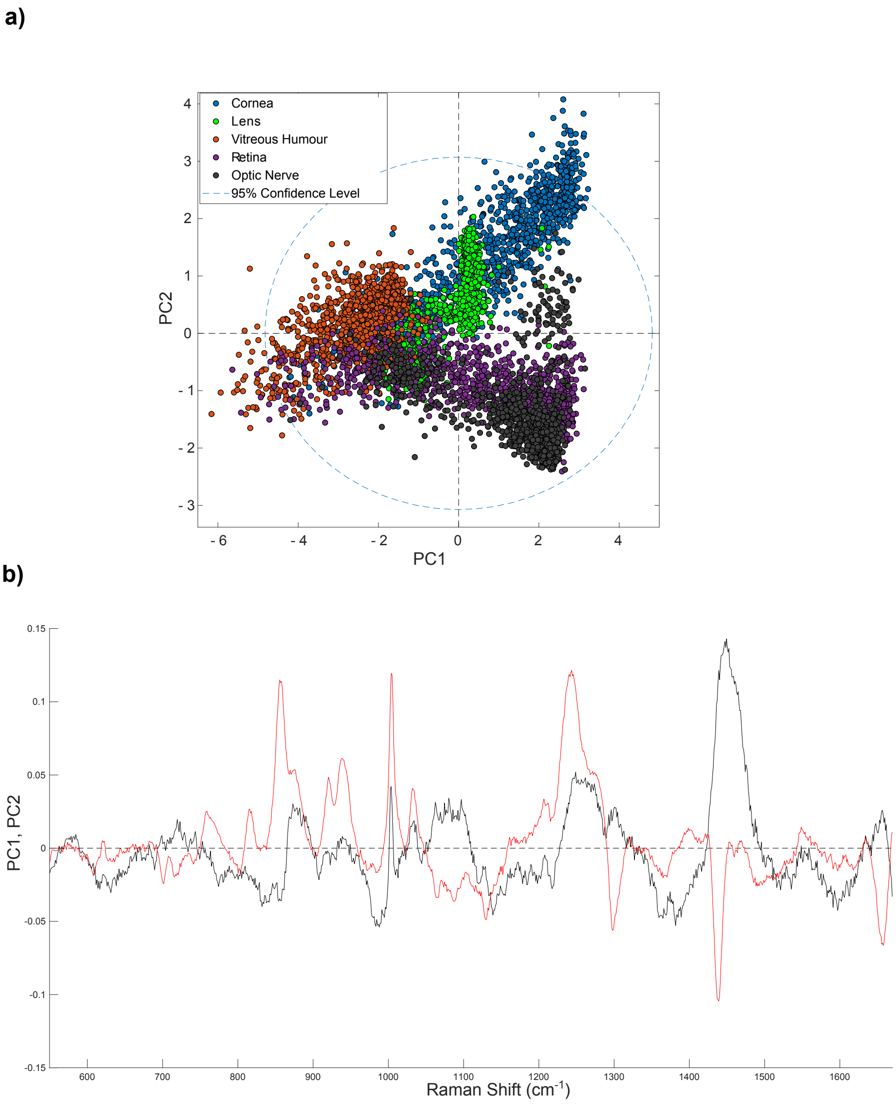

Figure S2: **a**, Scores plot for the first two principal components showing poor spatial separation of classes. **b**, Loadings for PC1 and PC2.

	Cornea	Lens	Vitreous Humour	Retina	Optic Nerve
Cornea	88.0	2.1	4.7	3.3	2.8
Lens	0.5	99.8	0.5	0	0.1
Vitreous Humour	1.0	0.1	96.5	2.7	0.5
Retina	1.0	0.2	2.3	95.7	1.7
Optic Nerve	4.6	0.71	0.8	2.2	92.5

Table S1: Confusion matrix showing average percentage for each class from the 1210 test spectra.

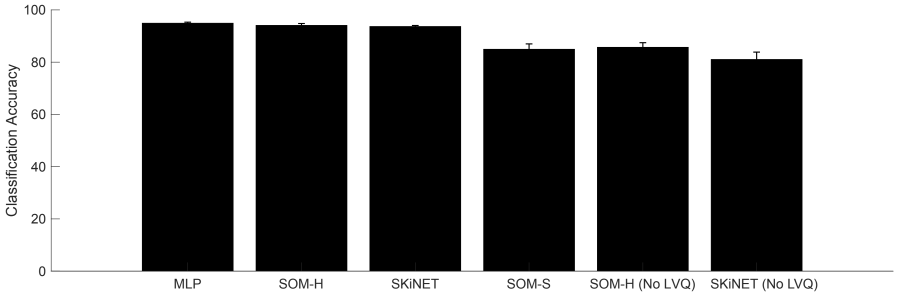

Figure S3: Comparison of classification accuracy for different approaches to SOM based classification. SOM-H referrs to using the hit count for class identification, SOM-S uses supervised SOMs as described here [16]
